# Trapped Esophageal Stent in a Child: An Unusual Complication

**DOI:** 10.1155/2020/8851112

**Published:** 2020-09-04

**Authors:** Mustafa Okumuş

**Affiliations:** Yeniyüzyıl University, Faculty of Medicine, Department of Pediatric Surgery, Gaziosmanpaşa Hospital and Bahat Hospital, İstanbul, Turkey

## Abstract

**Background:**

Migration is the most frequent and well-known complication of self-expandable metal stents (SEMS). Most of the time, migrated stents are still in the esophagus and can be relocated or removed successfully through endoscopy. However, what can be done if the stent is stuck between two esophageal strictures? Herein, we present a child with a trapped esophageal stent.

**Method:**

A 2-year-old male patient with an esophageal stent which migrated and became stuck between two esophageal strictures was reported.

**Results:**

Proximal stricture was excised, and the stent was removed via a right thoracotomy. Balloon dilatation was applied to the distal stricture. The patient was discharged on the 17th postoperative day without any problem.

**Conclusions:**

Pediatric patients with an esophageal stent should be closely followed up during this period. Early detection of complications makes treatment easier. Otherwise, there may be no option other than surgical treatment, as in the patient presented here.

## 1. Introduction

Migration is the most frequent and well-known complication of self-expandable metal stents (SEMS) [1]. Although several techniques for preventing stent migration and managing migrated stents have been described, stent migration remains a problem. When a SEMS migrates to the stomach, removal should be considered because of the risk of gastrointestinal obstruction and perforation. Most of the time, migrated stents are still in the esophagus and can be relocated or removed successfully through endoscopy. However, what can be done if the stent is stuck between two esophageal strictures? We have not encountered a similar case reported in the literature to date. Herein, we present a child with a trapped esophageal stent.

## 2. Case Presentation

**Figure 1:**
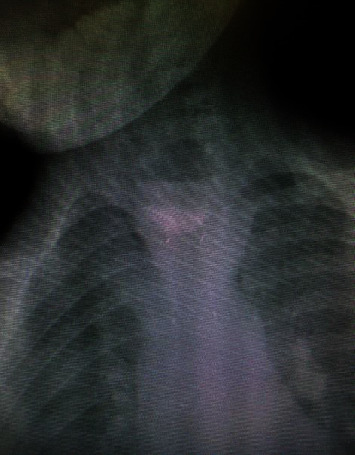
Esophageal graphy is showing no passage.

**Figure 2:**
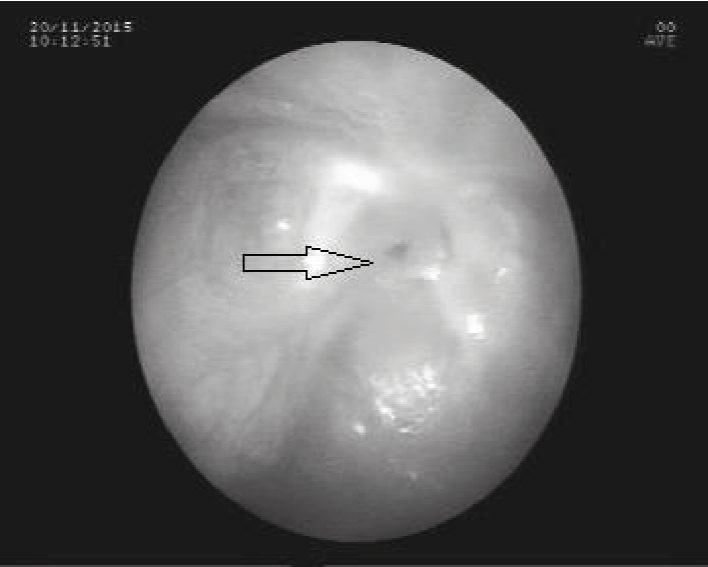
Almost a complete obstruction was revealed by endoscopy.

**Figure 3:**
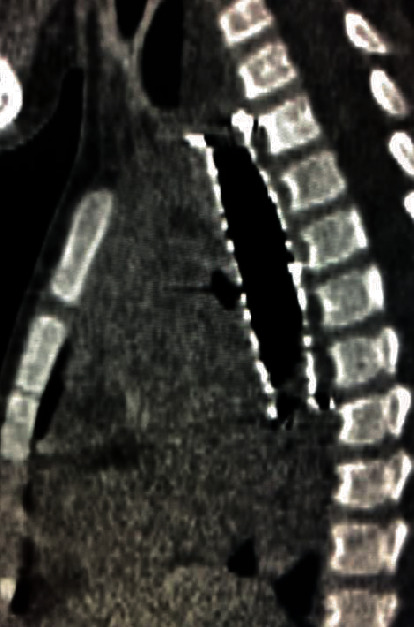
A SEMS under the esophageal stricture line.

A 2-year-old male patient from a foreign country was referred to our pediatric surgery clinic for esophageal replacement. According to the parent, esophageal stenosis developed a year ago due to corrosive ingestion. Balloon dilatation was tried first in the patient who had two strictures, one proximal and one distal. When endoscopic balloon dilatations failed for the proximal stricture, a SEMS was placed. The stent was repositioned after two weeks because of stent migration. In the meantime, the patient could not be followed up for three months due to the civil war in the country. During this time, the patient's nutritional status gradually deteriorated. In the last examination, it was revealed that the stent had migrated distally and the proximal esophagus was severely narrowed. It was thought that the stent could not be removed endoscopically. Gastrostomy was not considered, and the boy was referred for esophageal replacement. When the patient came to our clinic, he was fed with liquid food only, which took a long time, and he had difficulty swallowing his saliva. The patient was hospitalized, and total parenteral nutrition was started. Esophageal passage graphy showed almost no passage (Figure 1). When we performed endoscopy, we found almost complete obstruction (Figure 2). In tomography, it was revealed that the proximal esophagus was extremely widened and the stent was approximately 1 cm below (Figure 3). The lumen of the esophagus distal to the stent could not be evaluated. In the first stage, we aimed to excise the strictured part of the esophagus and remove the stent before the esophageal replacement. The strictured part of the esophagus was excised and the stent was removed via a right thoracotomy. On the fifth day, oral feeding was started and the thorax drain was removed on the 7th day. On the 14th day, the patient underwent endoscopy. The proximal esophagus was normal and balloon dilatation was applied to the distal stenosis. Distal stenosis was easily dilated. The patient was discharged on the 17th postoperative day without any problems. Now he is aged 4 years and doing well.

## 3. Discussion

The presence of various stent designs, each with different characteristics, has increased their use. Although the most common indication for esophageal stents is palliation of malignant dysphagia in adults [1], nowadays they are used for a wide variety of esophageal diseases, even in children [2]. Recalcitrant benign esophageal strictures due to corrosive ingestion is the most common indication for esophageal stents in children [2]. They are also used successfully in the management of postanastomotic esophageal strictures and leakages [3].

Early complications after esophageal stent placement include pain, bleeding, and perforation. Except for perforation, which is very rare, early complications are often not clinically relevant. Chest pain is the most common side effect after stent placement, but it is completely relieved within one or two days. The main problem is delayed complications, which are much more common. Delayed complications include stent migration (especially in covered stents), tumoral or mucosal ingrowths, bleeding, perforations, pressure necrosis, and blockage of the stents due to ingrowths [1–3]. Mucosal or tumoral ingrowths, which are frequently encountered in uncovered stents, make the removal of the stent significantly difficult.

Migration seems to be more common in fully covered stents, but it is a common complication of all stent types [4]. In general, the average migration rate is 29%, and the complication rate is 21% for all stent types [2]. Many new types of stents have been developed to prevent stent migration. The lower and upper ends of some stents have been made wider to prevent migration, and some have multiple protuberances on the outer surface for mucosal embedding [5]. In addition to the new designs of stents, additional methods have been introduced for stent fixation to prevent migration, such as Shim et al.'s method [6]. In the past few years, various attempts have been made to prevent stent migration by anchoring the stent to the esophageal wall, with clipping or endoscopic suturing devices. Retrospective case series have shown promising results, but migration rates are still reported as being between 11% and 17% [1].

Most stents have a purse-string wire loop at the upper flange, which facilitates stent repositioning or removal. If the SEMS is distally migrated but still in the esophagus, it can often be repositioned or removed easily. When a stent migrates to the stomach, some alternative techniques and devices can be used to deal with this situation such as an endoloop, polypectomy snares, and Sengstaken-Blakemore tubes [5, 7]. Although there are authors who recommend conservative follow-up, migrated stents should be removed whenever possible [8]. Conservative follow-up could be a good alternative in patients without a long life expectancy, but we think that this is not a reliable method for children.

The European Society of Gastrointestinal Endoscopy guideline on esophageal stenting recommends placing a fully covered SEMS and to keep it in place for at least 6-8 weeks to achieve optimal treatment response in benign strictures [1].

## 4. Conclusion

Pediatric patients in particular should be closely followed up during this period. Early detection of complications makes treatment easier. Otherwise, there may be no option other than surgical treatment, as in the patient presented here.
